# Ocular Tremor in Parkinson’s Disease: Discussion, Debate, and Controversy

**DOI:** 10.3389/fneur.2017.00134

**Published:** 2017-04-24

**Authors:** Diego Kaski, Adolfo M. Bronstein

**Affiliations:** ^1^Division of Brain Sciences, Department of Neuro-Otology, Imperial College London, London, UK; ^2^Department of Neuro-Otology, University College London, National Hospital for Neurology and Neurosurgery, London, UK

**Keywords:** eye oscillations, ocular tremor, Parkinson’s disease, vestibulo-ocular reflex, head tremor

## Abstract

The identification of ocular tremor in a small cohort of patients with Parkinson’s disease (PD) had lay somewhat dormant until the recent report of a pervasive ocular tremor as a universal finding in a large PD cohort that was, however, generally absent from a cohort of age-matched healthy subjects. The reported tremor had frequency characteristics similar to those of PD limb tremor, but the amplitude and frequency of the tremor did not correlate with clinical tremor ratings. Much controversy ensued as to the origin of such a tremor, and specifically as to whether a pervasive ocular tremor was a fundamental feature of PD, or rather a compensatory eye oscillation secondary to a transmitted head tremor, and thus a measure of a normal vestibulo-ocular reflex. In this mini review, we summarize some of the evidence for and against the case for a pervasive ocular tremor in PD and suggest future experiments that may help resolve these conflicting opinions.

## Introduction

Parkinson’s disease (PD) is a neurodegenerative condition characterized by motor features including bradykinesia, rigidity, tremor, and postural instability (1) and non-motor features such as anosmia, constipation, sleep disturbance, sexual impairment, cognitive impairment, and apathy (2). While the clinical phenotype in patients with PD may vary, a majority of patients will present with clinically appreciable tremor (3). The neural correlate of PD tremor has not been fully elucidated, but its generation appears to involve supraspinal oscillators within the cerebello–thalamo–cortical network (4–6). While PD tremor affects mostly the limbs, PD patients may have tremor of the tongue, lip, or chin (7). Early writings on PD state that head tremor is not a feature of PD, a view that remains commonly accepted, and a feature that differentiates PD tremor from essential tremor (8). Nevertheless, head tremor in PD has been described. A case series of five PD patients with head tremor revealed head tremor characteristics typical of PD limb tremor, including an increase in head tremor with mental calculation, disappearance during action, dopa-responsiveness, and similar tremor frequency to limb tremor (4–6 Hz) (9). In a single patient from this series, the authors performed electrophysiological recordings showing that the limb tremor and head tremor were coherent at their fundamental frequencies. The authors further ruled out mechanical conduction of the tremor by recording electromyography from the neck and arm muscles (9). It appears then that PD tremor may occur across different body segments independently, but simultaneously. In a separate report, a single PD patient was shown to have a tongue tremor and limb tremor of equal frequency (5 Hz) (10). Finally, Hunker and Abbs examined Parkinsonian rest tremor of the lips, jaw, tongue, and index finger in three PD patients, using electromyography (11). They found uniform resting tremor frequencies across orofacial and upper limb sites (11). Given that PD tremor can manifest at multiple sites simultaneously, and be synchronized, one question is whether ocular tremor might arise as a further tremor site in PD.

The wide availability of eye-tracking devices has seen a growth of research studies exploring oculomotor control across a range of clinical conditions, including PD. An early recording of ocular oscillations in a small cohort of patients with PD failed to receive further attention until a more recent report of a “pervasive ocular tremor” that was universally present in a cohort of 112 PD patients and mostly absent in healthy controls. Magnetic head tracking in a subset of patients did not reveal any tremulous head movements, implying that the observable ocular tremor was independent of head motion. Nevertheless, given the lack of other reports of ocular fixation instability across decades of eye movement recordings in patients with Parkinsonism, the possible origin of the pervasive ocular tremor generated significant discussion and controversy.

## Ocular Tremor in PD

Using infrared reflectometry, Duval and Beuter first described findings of ocular tremor in three out of five patients with PD (12). Ocular oscillations were mostly uniocular (in two patients), and in these localized ipsilateral to the side of the body most affected by PD. Those three patients had ocular oscillations of similar frequency to their resting limb tremor. There was no relationship, however, between the amplitude of the eye oscillations and amplitude of resting limb tremor. In these patients with asymmetrical eye oscillations, the presence of square wave jerks in both eyes, equal in amplitude, meant that the monocular nature of the oscillation could not be attributed to an artifact of scaling between the two eyes. Patient’s head was stabilized by asking patients to bite onto a wooden tongue depressor, attached to the chair on which they sat for the eye movement recordings. The authors argued that the ocular tremor was due to an “attractor effect” on movement related to the generator of the limb rest tremor, which would explain why the frequency of the ocular tremor in their three PD patients was similar to the frequency of the rest tremor of the limbs (13). Nevertheless, it is possible that such an ocular tremor may have a neural oscillator that is independent to the limb oscillator, particularly as coherence values from tremor data from each eye with rest tremor were different for two of the five PD patients. Uniocular tremor has been reported following an isolated olivary nucleus lesion (14).

## “Pervasive Ocular Tremor” in PD

**Figure 1:**
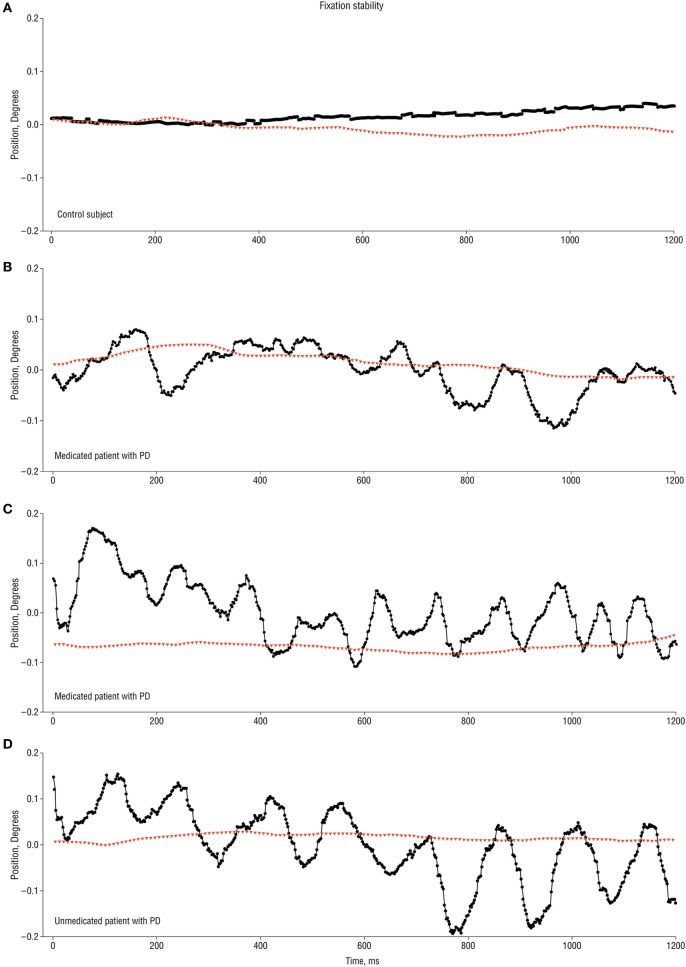
**1.2 s recording traces of ocular tremor in patients with Parkinson’s disease (PD) and healthy control [from Ref. (15) with permission]**. **(A)** Eye movement recording from a control subject showing stable fixation (no ocular tremor). **(B,C)** Two different medicated PD patients showing eye oscillations of variable amplitude and approximately 6 Hz **(B)** and 10 Hz **(C)**. **(D)** Ocular tremor in an unmedicated PD patient with a larger amplitude eye oscillation of approximately 10 Hz frequency. Note the absence of any head tremor in all traces. Black circles represent horizontal eye movements, with positive values indicting rightward eye movements, and red triangles indicating rotational head movement along the azimuth.

Gitchel and colleagues studied the eye movements of 112 patients with idiopathic PD during steady fixation (15). They identified a continuous oscillatory fixation instability that they termed “pervasive ocular tremor” in *every* PD patient. The oscillations had an average fundamental frequency of 5.7 Hz (i.e., within the range of the limb tremor in PD; 4–7 Hz), a mean horizontal amplitude of 0.27°, and mean vertical amplitude of 0.33°. The tremor persisted for the duration of the recording, although the waveform characteristics were variable (15). Figure 1 taken from the original manuscript shows a typical 1.2 s recording of the ocular tremor. The authors did not use head restraint but recorded head movements using a magnetic tracker in a subset of 62 PD patients and 31 controls; no head oscillation was detected in any subject.

## Pathophysiology of Ocular Tremor

In order to try to understand the possible neural mechanism underlying the observed ocular tremor (fixation instability) in PD, one might first consider the necessary requirements of the oculomotor system to maintain steady fixation. Thus, the fixation target must rest upon the retinal fovea—an area of approximately 0.5° in diameter with the highest visual acuity (16). In addition, the image must not move more than approximately 5°/s across the retina (17), otherwise the subject would experience oscillopsia (illusory motion of the visual world). When the head is free, steady fixation thus requires the vestibulo-ocular reflex, which compensates for head movements by generating an eye movement of the same velocity but opposite direction to the head movement. If the vestibulo-ocular reflex is absent, even cardiac pulsations transmitted to the head can disrupt vision (18). When the head of a healthy subject is immobilized (e.g., with a bite bar), the subject’s gaze does not in fact remain completely still but is disrupted by microtremor, microsaccades, and ocular drifts (19, 20). Therefore, could the “pervasive ocular tremor” observed in patients with PD be one of these types of eye movements?

Microtremor has a mean frequency of approximately 84 Hz and ranges from 70 to 103 Hz. Due to its high frequency and very small amplitude (1 photoreceptor width, <0.5 arcmin), microtremor does not disrupt vision (21). Microsaccades are rapid movements with frequencies of 1–2 Hz, and typically less than 1° in size. They are thought to prevent perceptual fading during fixation (22, 23). Recent work suggests that square wave intrusions, a common finding in patients with neurodegenerative movement disorders, lie on a continuum with microsaccades (24, 25). Smooth intersaccadic drifts during attempted fixation are thought to be under the control of smooth eye movements (26) and typically do not exceed 0.1°/s, unless visual feedback is removed, for example, by switching from a light to a dark environment (27). Thus, despite all these eye movements that occur during steady fixation, the SD of gaze is typically <0.2°, so while the eyes are not perfectly still, the image of interest stays mostly over the fovea, and image motion is not perceptible. Thus, given the characteristics of the “pervasive ocular tremor” in PD, they cannot be considered microtremor, microsaccades, or ocular drifts.

In contrast to these physiological “fixation ocular movements,” blurred vision and oscillopsia occur when abnormal eye movements, such as acquired pendular nystagmus, cause movement of the retinal image greater than exceeds 5°/s (21). Gitchel and colleagues in fact commented that the pervasive ocular tremor observed in patients with PD was reminiscent of pendular nystagmus given the sinusoidal waveform and similar fundamental frequencies (15). They acknowledge, however, several notable differences to pendular nytsagmus, such as the smaller amplitude of the waveform they reported in their PD patients, compared to that normally seen in pendular nystagmus. Moreover, in pendular nystagmus, the phase of the oscillations is reset by saccades and this was not the case in the ocular tremor described in PD patients. Most importantly perhaps, acquired pendular nystagmus causes oscillopsia, and this was not reported by any of the PD patients studied by Gitchel et al. (15).

Finally, it is conceivable that the pervasive ocular tremor in PD stems from subtle head oscillation, inducing a normal VOR response, causing an oscillation of the eyes in response to head movement. We now summarize the evidence in support of a pervasive ocular tremor, and the evidence in support of apparent ocular tremor resulting from head oscillation (and an intact VOR).

## Evidence in Support of a Pervasive Ocular Tremor Inherent to PD

1. In their original description of ocular tremor in patients with PD, Duval and Beuter did not find a systematic or direct relationship between fluctuations in rest tremor of the hand and fluctuations of eye movement amplitude during ocular fixation in patients with PD (12), suggesting that the ocular tremor was independent of the limb rest tremor.
2. Duval and Beuter asked their PD subjects to bite onto a tongue depressor attached to the structure of the chair to stabilize the head during eye in space ocular movement recordings (12). The authors, however, acknowledged the possibility that head movement occurred (an accelerometer was not used to record head movements), resulting in activation of the vestibulo-ocular reflex, that could in turn explain the eye oscillations. One wonders whether asking patients to bite onto a wooden tongue depressor may itself generate a head or jaw tremor. Nevertheless, this would not explain why the oscillations were so asymmetrical between the two eyes. Moreover, the fact that all 112 PD patients in the cohort from Gitchel et al. displayed a recordable eye tremor, including unmedicated patients, suggests that ocular tremor may be a fundamental property of PD (15). Moreover, only 2 of 60 healthy controls in the study by Gitchel et al. were also found to have an ocular tremor despite no signs of Parkinsonism, one of whom then developed PD within 3 years of follow-up (15).
3. A magnetic tracker was employed to evaluate the possible contribution of head motion to the presence of the ocular tremor. The authors, however, consistently recorded no head tremor in a subset of 62 PD patients (who did, however, have ocular tremor). Electromagnetic motion recorders provide accurate displacement measures for large-amplitude, low frequency movement. Conversely, they are less accurate for low amplitude, high frequency movement, for which accelerometers are superior (28). In this light, Gitchel et al. recorded eye movements in a further subset of eight patients (29), during simultaneous head movement recording using both a triaxial accelerometer and electromagnetic tracker. They again failed to record any appreciable head tremor, despite evidence of continuous ocular tremor.
4. Indeed, ocular tremor was observed in this group of eight PD patients irrespective of whether the head was free or fixed (by mean of a head holding device and a dental impression bite plate), implying that head motion had no effect upon the presence or magnitude of the ocular tremor (29).
5. Gitchel et al. found no relationship between the amplitude of ocular tremor and clinical rating of arm tremor across their total cohort (15). This suggests that ocular tremor occurs independently of appendicular tremor and hints at a different neural generator for the tremor. Similarly, many patients from their large PD cohort had no appreciable appendicular tremor.
6. A previous study in patients with essential tremor (who often manifest head tremor) found no evidence of ocular instability, further suggesting a decorrelation between head tremor and ocular tremor (30). In this study, head tremor was assessed using a magnetic tracking device and was only apparent in two patients with ET. This is surprising given the prominent head tremor in ET patients (8).

## Evidence in Support of Apparent Ocular Tremor Resulting from Head Oscillation

Refuting the non-existence of a proposed clinical sign poses inherent challenges, all the more so in the case of a Parkinsonian ocular tremor that has never been observed clinically nor contributes to any visual disability in these patients. Scientific studies in small numbers of patients have suggested that the ocular tremor described in patients with PD may indeed be related to head oscillations, indicative of an intact VOR.

**Figure 2:**
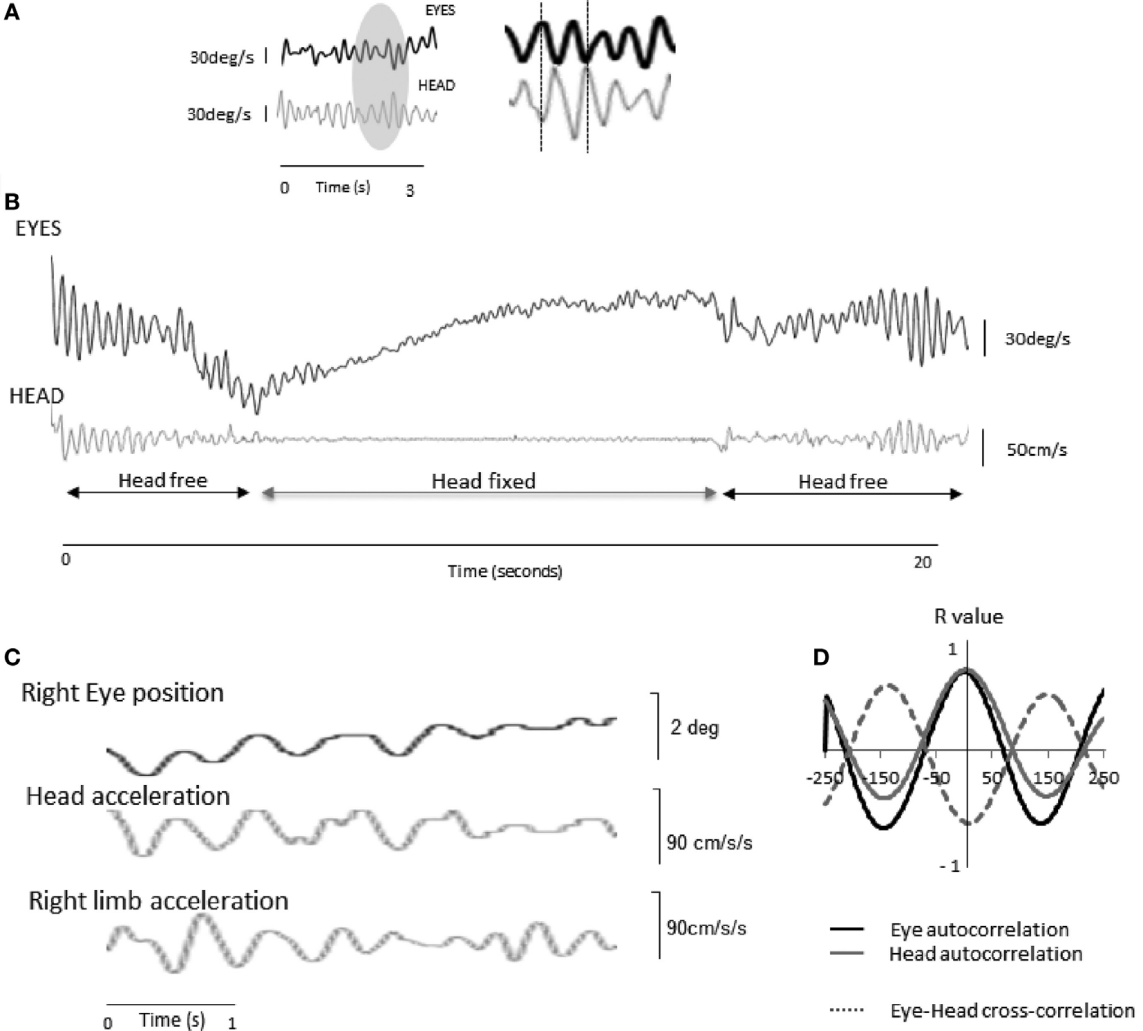
**Eye and head oscillations in two patients with Parkinson’s disease (PD) [taken from Ref. (36) with permission]**. **(A)** Patient 1—a 3 s recording of the eye (infrared video-oculography) and head (accelerometry) movements in a patient with PD with the head free.* To the right, a magnified view of the recordings reveals that the ocular oscillation is in antiphase to the head tremor. **(B)** When the head is physically restrained, the ocular tremor decreases in amplitude, indicating that the ocular tremor is intrinsically linked to the head tremor, and thus part of an intact VOR response. The ocular tremor is not abolished as the head can never be completely immobilized. **(C)** Raw oculographic, head, and limb tremor traces in a separate PD patient, without head restraint. The amplitude of the head and ocular tremor was smaller than in patient 1, in keeping with a smaller right limb tremor. Head tremor was not clinically visible in this patient. Head and limb oscillations were recorded from linear accelerometers. The eye and head traces appear in phase as a result of 180-degree phase shift between position (eye) and acceleration (head). **(D)** Autocorrelations for the eye and head traces and cross-correlation between the two signals over a 500-ms window for a fundamental frequency of 4.5 Hz. R values are shown in the *y* axis. *NB: In **(A)** and **(B)** head and eye traces are expressed in angular velocity units. Head acceleration values were, therefore, digitally integrated (linear acceleration to linear velocity), and corrected for eccentricity (tangential linear velocity to angular velocity, by taking into account occiput to head rotational axis distance, approximately 10 cm) using standard equations. As a leftward head rotation induces rightward occiput motion, the accelerometer trace has been inverted to correct for polarity. Finally, eye displacement recordings have been digitally differentiated (degrees to degrees/s).

1. Apart from the early report by Duval and Beuter (12), and despite extensive oculographic recordings in PD, ocular tremor had not previously been described. In fact, Leigh and colleagues looked back through early oculomotor recordings in PD—using the goldstandard scleral search coil technique—and were not able to identify any evidence of ocular tremor in their studies of Parkinsonian patients over the past 30 years (31, 32), presumably because they used a chair-fixed restraint to stabilize the patients’ heads (thus significantly reducing head oscillations).
2. Patients with head tremor and bilaterally *impaired VOR* have eye oscillations on fundoscopy (eye in space oscillations as there is a shift in gaze without any movement of the eyes relative to the head), termed pendular pseudonystagmus (33–35) of similar amplitude to that reported by Gitchel et al. (15). In this condition, gaze stability is negatively affected by the head tremor due to the insufficient vestibularly mediated compensatory eye movements. Patients, therefore, report oscillopsia and the clinician can observe oscillation of the fundus during ophthalmoscopy. The fact that most patients with head tremor do not report oscillopsia, nor do they show oscillation of the fundus, is testimony to how exquisitely tuned the VOR is to generate high frequency compensatory eye movements. For this reason, a well know artifact in eye movement recordings in patients with head tremor is an apparent oscillation of the eye (eye-in-orbit as there is no shift in gaze with a normal VOR) in the oculographic trace, which is reduced during forced immobilization of the head. However, it is also known that complete immobilization of the head in human patients with significant head tremor is extremely challenging; both in patients with essential (33) and Parkinsonian tremor of the head (36), a very significant reduction of the head and eye oscillation can be obtained but complete elimination is rarely feasible.
3. Eye movements were recorded in two consecutive PD patients attending an eye movements and balance clinic. These patients also underwent simultaneous recording of head and limb movements using an android application triaxial accelerometer (36), with comparable resolution to standard axial accelerometers (37). Despite different limb tremor amplitudes in these patients, ocular oscillations were identified in both patients. These were accompanied by a recordable head tremor that had the same fundamental frequency and high coherence with both the eye oscillation and a recordable limb tremor (Figure 2). The eye oscillations were in the opposite direction (antiphase) to the head oscillation and dampened by physically restraining the head (36). This suggests that the ocular tremor is a compensatory eye movement secondary perhaps to a head tremor transmitted from the limb. The fact that these findings led to opposite conclusions to the study by Gitchel et al. raises important questions about the technical aspects of the data acquisition and patient population studied. Gitchel and colleagues used a magnetic tracker device rather than an accelerometer to record head movements; Kaski and colleagues suggest accelerometers are superior to magnetic tracking devices to record low amplitude high frequency tremor. In their follow-up study, Gitchel and colleagues used both a triaxial accelerometer and magnetic tracking device to record head tremor in eight PD patients. They found a “complete (three dimensional) lack of head movement” in these patients. Given the similarities in the accelerometers employed, the lack of head movement may relate to the use of individualized bite plates to avoid head motion. Nevertheless, such a finding remains interesting given the difficulty in achieving complete head stabilization, even with the use of bite plates, in some oculographic studies in patients with prominent head tremor (33).
4. A notable difference between the ocular tremor of PD and other known eye movements is that the pervasive ocular tremor was reportedly unaffected by “saccades, blinks, or other eye movements” (15). In contrast, every other form of ocular oscillation has been reported to be “influenced by saccades, gaze angle, convergence, or vestibular stimuli” (21). This would, therefore, suggest that the source for PD pervasive ocular tremor lies outside of the ocular motor system, such as head tremor.
5. Involuntary eye oscillations, such as in patients with acquired pendular nystagmus or downbeat nystagmus syndrome, cause troublesome oscillopsia (21). The small amplitude of the pervasive ocular tremor recorded in PD patients may have been too small to displace the fixation target from the fovea, but their high frequency would cause their root mean square velocity to exceed 5°/s, which would be expected to induce oscillopsia (38, 39). One might argue that a large root mean square retinal slip value could be accounted for by a few extreme peak values during visual fixation, whereby the majority of the eye movements are within a tolerable low-velocity range that would allow for stable vision. It has been shown, however, that root mean square velocity values of retinal slip are of clinical relevance to gaze stabilization (39).
6. A continuous eye oscillation at approximately 5 Hz and approximately 0.3° (as in “pervasive ocular tremor”) should be visible during direct ophthalmoscopy (40), which amplifies the retinal image by upto a factor of 15. Indeed, ophthalmoscopy is a sensitive method for detecting eye movements (14), even as small as 0.1° [e.g., microflutter (41)]. The pervasive ocular tremor described by Gitchel et al. (15) should, therefore, be visible with an ophthalmoscope and this has never been reported in the literature. In response to this, Gitchel and colleagues oscillated a prosthetic eye using a galvanometer motor over a range of amplitudes and frequencies but did not observe oscillations of the scleral vessels at very small amplitudes (42). The properties of a prosthetic eye (and in particular the ocular media) are inherently different to the living eyeball, and therefore, further fundoscopic studies in PD patients are required to clarify this issue.
7. Duval and Beuter described uniocular tremor in patients with PD (12), which is different to the bilateral ocular tremor reported by Gitchel et al. (15). Uniocular microtremor has been seen in a patient with asymptomatic oculopalatal tremor secondary to haemorrhagic injury affecting the inferior olivary nucleus (14). This raises the question of whether the uniocular tremor described in patients with PD by Duval and Beuter had pathology affecting the inferior olivary nucleus, rather than the tremor being a fundamental feature of PD.

## Clinical Implications

Because it was posited that the pervasive ocular tremor might be a clinical biomarker for PD, including in the diagnosis at the pre-symptomatic stage, it is important to scrutinize the findings of Gitchel et al. before the presence of ocular tremor gains wide acceptance as a biomarker for PD. Interestingly, electromyography has revealed rhythmical muscle activity in patients with PD despite no clinical tremor (43). This suggests that there may be a subclinical tremor in patients with PD, and that such a tremor could be transmitted to the head and manifest as an ocular tremor if the head is not fixed. Further work is needed to identify whether head tremor is indeed a ubiquitous finding in patients with PD, irrespective of clinical tremor, and whether such tremor could be identified using eye movement recordings. In such case, one would also need to find an explanation for the absence of oscillopsia in PD patients.

## Future Directions

Further research from different laboratories is warranted to investigate whether patients with PD do indeed show impaired visual fixation behavior, and to systematically study the characteristics of any ocular tremor that might be detected. For such a study, eye movements should be recorded using high-resolution techniques such as scleral search coil, infrared eye tracking, or video-oculography. The head should ideally be immobilized using a custom-made bite bar, or the eye tracker should be insensitive to movements of the device with respect to the subject’s head (as is available on several modern eye trackers). There should be simultaneous recording of the eyes, head, and distal limb, ensuring that the devices are appropriately calibrated, and the signals are synchronized. Tremor recording of the head and limbs should be performed using accelerometers rather than position tracking devices. Signals should, therefore, be acquired at the same sampling rate and in the same plane (e.g., yaw and pitch planes). Tremor frequency, amplitude, and coherence between tremor sites should be assessed. Such a study would benefit from including patients with a range of tremor syndromes and in PD preferably include assessments after the withdrawal of medication to exclude possible dopaminergic-related effects.

## Author Contributions

DK reviewed the literature and compiled the manuscript and figures. AB reviewed the literature and approved the final version of the manuscript.

## Conflict of Interest Statement

The authors declare that the research was conducted in the absence of any commercial or financial relationships that could be construed as a potential conflict of interest.
